# ‘Beyond “test and treat” – malaria diagnosis for improved pediatric fever management in sub-Saharan Africa’ by Emily White Johansson

**DOI:** 10.3402/gha.v9.34416

**Published:** 2016-12-16

**Authors:** S. Patrick Kachur

**Affiliations:** Malaria Branch, Division of Parasitic Diseases and Malaria, Center for Global Health, US Centers for Disease Control and Prevention, Atlanta, GA, USA

Since 2010, the World Health Organization has recommended universal malaria diagnostic testing to guide treatment of febrile illness as a fundamental component of malaria case management (1). Malaria control programs and their partners have been gradually expanding diagnostic testing, largely through point-of-care rapid diagnostic tests (RDTs). For the past 2 years, global purchases of malaria RDTs have equaled or slightly exceeded treatment doses of artemisinin-based combination therapies (ACT), and nearly half of globally reported malaria cases have been diagnostically confirmed (2). Emily White Johansson's doctoral thesis – summarized in this issue (3) – examined early experience expanding malaria diagnostic testing across sub-Saharan Africa and points to some key directions for additional scholarship and program implementation.

Johansson and her colleagues (4–7) interrogated nationwide malaria coverage surveys from more than a dozen countries, conducted original qualitative and quantitative research in Uganda, and unleashed powerful big data approaches on a comprehensive health service assessment data set from Malawi – all with the goal of expanding the previously limited understanding of malaria diagnostic practices just as malaria RDTs were being introduced for routine practice. Collectively, it is an analytical *tour de force*. The resulting publications demonstrate program-relevant observations. Access to malaria diagnostic testing was poor and wildly inequitable shortly following the universal diagnosis policy – particularly in peripheral health facilities. None of the nationwide surveys showed the promised reductions in ACT consumption at a population level, at least not at first. Moreover, countries varied greatly in terms of what impact malaria diagnostic testing had on fever management overall. While clinical guidelines for antibiotic use appeared straightforward, they still required considerable judgment on the part of health workers – who were frequently left feeling unsupported, especially when malaria test results were negative. Once malaria diagnostic testing had become established, however, a majority of health workers adapted to using the results to guide malaria-specific treatment. But antibiotic overtreatment was common, especially among children testing negative for malaria, and was influenced by the presence of other symptoms such as cough or difficult breathing. Arriving at each of these discrete findings, Dr. Johansson establishes an approach that she and others can revisit as new evidence and experience accumulate.

While each of the four specific papers stands on its own, Johansson's skill really shines in the front matter and discussion that bring these disparate approaches into sharp focus and point toward program action. The works are linked by a thoughtful conceptual framework that emphasizes access, facility readiness, and clinical practice. The diffusion of innovations theory affords a useful perspective from which Johansson and her colleagues consider their findings in light of rapid and ongoing changes in policy and practice. She also contextualizes malaria RDT implementation in the integrated management of childhood illness and challenges the dominant focus on malaria-related commodities and outcomes. She ends by advancing the case that malaria diagnostics represent an underappreciated opportunity for malaria-specific investments to contribute to broader systems strengthening efforts.

The implications of Johansson's studies for future work are varied. There is still much to be done before malaria diagnosis becomes universally available and applied. This will require a further commodity shift so that RDTs can greatly outnumber ACT treatments. But there is good evidence now that despite some initial concerns health workers can quickly adjust to making malaria treatment decisions on the basis of a diagnostic test. The situation is far less clear for the management of non-malarial fevers, and more thought is needed to rationalize treatment decisions and antibiotic prescribing in this setting. It would also be troubling if the fledgling confidence in malaria RDTs were undermined by concern over focal reports of malaria parasite populations undetectable by the most common testing platform (8). The global community should prioritize careful surveillance for this phenomenon and the development, production, and deployment of tests based on more stable markers. In addition, much of Johansson's analyses are based on data collected when malaria diagnostic tests were still limited to hospitals and health centers, but programs are expanding RDTs to peripheral facilities, community health workers, and even private retail outlets. It would be useful to update some of the analyses with more current data sets.

Finally, malaria policy makers would be well-advised to accept Dr. Johansson's challenge and recognize the important opportunity universal malaria diagnostic testing presents for enhancing health systems through malaria investments. This should include a more comprehensive program to address children who test negative, including the right guidance, commodities, and support to empower health workers to treat them adequately. Another important direction would be to ensure that the information created by wider application of malaria RDTs was captured and reported through improved surveillance systems. Johansson's title only partially references WHO's T3 strategy for scaling up malaria diagnosis: test, treat, and *track* (9). Capturing surveillance information on diagnostically confirmed malaria cases would generate the data to guide malaria control investments and prepare the way for ambitions of elimination. Similarly, tracking non-malarial illnesses could be a key to identifying other infectious diseases of epidemic potential.

Global policy and procurements for malaria diagnostics still lean heavily toward the disease-specific Millennium Development Goals. By encouraging the malaria community to embrace the systems strengthening potential of its investments, Dr. Johansson also points us toward closer alignment with the more cross-cutting elements of the Sustainable Development Agenda. Let's take her up on it.
